# Supplementary material for: Health outcomes in Deaf signing populations: A systematic review
Source: PLoS One. 2024 Apr 16;19(4):e0298479. doi: 10.1371/journal.pone.0298479 (PMC11020444; doi:10.1371/journal.pone.0298479)
Supplement: S1 Appendix — (PDF) [file pone.0298479.s001.pdf]

A systematic review of evidence concerning health in Deaf signing populations

Review methods were amended after registration. Please see the revision notes and previous versions for detail.

Citation

Katherine Rogers, Aleix Rowlandson, James Harkness, Gemma Shields, Alys Young. A systematic review of evidence concerning health in Deaf signing populations. PROSPERO 2020 CRD42020182609 Available from: https://www.crd.york.ac.uk/prospero/display_record.php?ID=CRD42020182609

Review question [2 changes]

The primary review question is: What does the available literature conclude about the mental and physical health of the adult Deaf population?

The secondary review questions are:

• (If data available) How does the health of the adult Deaf population compare to that of the general population?

• What are the strengths and weaknesses of the available literature?

• What should future research aim to address?

Searches [2 changes]

We will search the following electronic bibliographic databases in order to identify studies for the review:

• MEDLINE

• Embase

• PsycINFO

• Web of Science

The search strategy will be a search of “(deaf* OR hearing loss*) AND sign* AND (health* OR wellbeing*)”. Searches will not be restricted by language.

Types of study to be included [2 changes]

Identified studies will be screened using explicit inclusion and exclusion criteria.

The inclusion criteria are:

• All study types other than those in the exclusion criteria

• Method focused papers which also contain results in relation to the health of the Deaf signing population

• Full-text articles, published in peer-review journals

The exclusion criteria are:

• Papers focused entirely on methods rather than health

• Editorial pieces

• Letters

• Historical articles

• Conference abstracts – full text must be available

• Unpublished theses

Condition or domain being studied [4 changes]

The term Deaf (capital D) usually refers to deaf people who are sign language users, distinguished from those who have hearing loss and use spoken language and/or have lost their hearing later in life. In the same way as any language-using population, a distinct culture is associated with their language use (Deaf culture) which has both national and trans-national features. Most Deaf people were born deaf or became so in early childhood but have hearing parents. However as cultural Deaf identity and sign language use is for many a choice and/or cultural affiliation, there are growing numbers of young Deaf people who are spoken language users first and come to sign language as a second language later in childhood. Levels of literacy in written word remain low in Deaf populations globally at the same time as access to knowledge and information in signed languages remains highly restricted. This is known to impact on health literacy amongst Deaf people. Health inequalities in terms of health outcomes and access to services have also been noted previously in the literature. However, a comprehensive and systematic review of the evidence concerning physical and mental health amongst Deaf people has yet to be undertaken.

Participants/population [3 changes]

The inclusion criteria are:

• The signing Deaf population (using a sociological definition, referring to a particular cultural-linguistic group, not defined by the audiological condition)

• Adults (using the ages defined within the literature)

• Signing populations from any country who use their national sign language(s) by preference or as their only language

The exclusion criteria are:

• deaf and hard-of-hearing populations who are not sign language users

• Individuals who are blind/ those with dual sensory impairment (deafblind) and who do not use a signed language

• Populations solely consisting of Deaf adolescents or children (using the ages defined within the literature)

Intervention(s), exposure(s)

Exposure - a member of the Deaf population.

Comparator(s)/control

None.

Main outcome(s) [3 changes]

The outcome we will explore is the health of the Deaf population, both mental and physical. This will include measures of mental health, such as the prevalence of mental health conditions and measures used in relation to mental health (e.g. the PHQ-9 and GAD-7). It will also include measures of physical health, such as the prevalence of chronic conditions and symptom measures for physical health conditions. Furthermore, it will include measures of overall health status, quality of life and wellbeing, that may reflect mental and physical health combined.

Measures of effect

As we expect there to be limited evidence, and we are uncertain of what data we will identify, we wish to keep the outcome measures broad in order to give a comprehensive overview of the state of wellbeing in the Deaf population as possible.

Additional outcome(s) [3 changes]

As we expect there to be limited evidence on this subject, we also aim to analyse the quality of the available literature in order to help improve future research.

Measures of effect

The quality of the studies will be measured using the appropriate (according to study type) Critical Appraisal Skills Program (CASP) tool.

Data extraction (selection and coding) [4 changes]

Potentially relevant research articles identified from the database searches will be downloaded and uploaded to Rayyan (systematic review software) for review. Titles and abstracts of the studies retrieved from the pre-specified search strategy will be screened during the primary screening stage. Full texts of potentially eligible studies will then be screened during the secondary screening stage. Screening will be done using the inclusion/exclusion criteria outlined. A list of studies to be included will be formulated and the search flow will be presented in a PRISMA diagram in any dissemination activities. Each stage of the review will be completed independently by two reviewers, with a third reviewer consulted to settle any disagreements.

Comprehensive data extraction will be performed using a pre-specified data extraction tool developed using the Cochrane data collection form (available here: https://dplp.cochrane.org/data-extraction-forms) and other data extraction forms used by the research team. This includes extracting information on study samples, methodology, limitations, evidence gaps, results, and a quality assessment for critical appraisal. Three reviewers will extract data independently and any queries or issues will be discussed and decisions reached unanimously by the whole review team.

Risk of bias (quality) assessment [2 changes]

CASP checklists will be used to assess for bias in each study involved in the analysis. The relevant checklist will be chosen according to the study design.

These will be accessed from https://casp-uk.net/casp-tools-checklists/

Second reviewers will also be involved in the assessment which will reduce the risk of bias in this review.

Strategy for data synthesis [2 changes]

A narrative synthesis of findings will be presented as it is unlikely that sufficient data will be identified to conduct a meta-analysis (the authors anticipate that a limited evidence base reporting a variety of study designs and measures of health will be identified). Tables will be used to clearly report key features of each eligible study. The narrative will be structured around key features of study design and main results; including year of publication, country of study, study design, target population and sample, comparison population(s) (if included in study), outcomes reported that are relevant to physical and mental health and key findings. A bias table will also be presented (using the appropriate CASP checklist for each study type) to summarise the bias assessment for each study. The Synthesis without meta-analysis (SWiM) in systematic reviews reporting guidelines will be adhered to (including discussion on the limitations of the synthesis).

Analysis of subgroups or subsets [1 change]

Dependent on the availability of information on subgroups - analysis will be decided on later

Contact details for further information

Katherine Rogers

katherine.rogers@manchester.ac.uk

Organisational affiliation of the review

University of Manchester

Review team members and their organisational affiliations [2 changes]

Dr Katherine Rogers. The University of Manchester

Ms Aleix Rowlandson. University of Manchester

Dr James Harkness. University of Manchester

Ms Gemma Shields. The University of Manchester

Professor Alys Young. The University of Manchester

Type and method of review

Narrative synthesis, Prognostic, Systematic review

Anticipated or actual start date

17 March 2020

Anticipated completion date [3 changes]

01 December 2022

Funding sources/sponsors [1 change]

This review is partly funded by Dr Katherine Rogers's NIHR Post-Doctoral Fellowship (NIHR award reference number: PDF-2018-ST2-004). The views expressed in this publication are those of the author(s) and not necessarily those of the NIHR, NHS or the UK Department of Health and Social Care.

Conflicts of interest

Language

English

Country

England

Stage of review

Review Ongoing

Subject index terms status

Subject indexing assigned by CRD

Subject index terms

Deafness; Humans; Persons With Hearing Impairments

Date of registration in PROSPERO

10 August 2020

Date of first submission

27 April 2020

Stage of review at time of this submission [5 changes]

**Stage**	**Started**	**Completed**
Preliminary searches	Yes	Yes
Piloting of the study selection process	Yes	Yes
Formal screening of search results against eligibility criteria	Yes	Yes
Data extraction	Yes	Yes
Risk of bias (quality) assessment	Yes	Yes
Data analysis	Yes	Yes

Revision note

Updated stage of review to completed

The record owner confirms that the information they have supplied for this submission is accurate and complete and they understand that deliberate provision of inaccurate information or omission of data may be construed as scientific misconduct.

The record owner confirms that they will update the status of the review when it is completed and will add publication details in due course.

Versions

10 August 2020
30 March 2022
29 April 2022
29 April 2022
13 May 2022
22 June 2022
07 September 2022
21 September 2022
01 November 2022
27 February 2024
