# Supplementary material for: Health outcomes in Deaf signing populations: A systematic review
Source: PLoS One. 2024 Apr 16;19(4):e0298479. doi: 10.1371/journal.pone.0298479 (PMC11020444; doi:10.1371/journal.pone.0298479)
Supplement: S1 Table — (DOCX) [file pone.0298479.s003.docx]

**S1 Table: keywords used in the search strategy**

| **No.** | **Search term** |
| --- | --- |
| 1 | Deaf*.mp. [mp=ti, ab, hw, tc, id, ot, tm, mh, tn, dm, mf, dv, kf, fx, dq, nm, ox, px, rx, ui, sy] |
| 2 | Sensorineural hearing loss*.mp. [mp=ti, ab, hw, tc, id, ot, tm, mh, tn, dm, mf, dv, kf, fx, dq, nm, ox, px, rx, ui, sy] |
| 3 | 1 or 2 |
| 4 | sign*.mp. [mp=ti, ab, hw, tc, id, ot, tm, mh, tn, dm, mf, dv, kf, fx, dq, nm, ox, px, rx, ui, sy] |
| 5 | health*.mp. [mp=ti, ab, hw, tc, id, ot, tm, mh, tn, dm, mf, dv, kf, fx, dq, nm, ox, px, rx, ui, sy] |
| 6 | wellbeing*.mp. [mp=ti, ab, hw, tc, id, ot, tm, mh, tn, dm, mf, dv, kf, fx, dq, nm, ox, px, rx, ui, sy] |
| 7 | 5 or 6 |
| 8 | 3 and 4 and 7 |
| 9 | letter*.mp. [mp=ti, ab, hw, tc, id, ot, tm, mh, tn, dm, mf, dv, kf, fx, dq, nm, ox, px, rx, ui, sy] |
| 10 | editorial*.mp. [mp=ti, ab, hw, tc, id, ot, tm, mh, tn, dm, mf, dv, kf, fx, dq, nm, ox, px, rx, ui, sy] |
| 11 | case report*.mp. [mp=ti, ab, hw, tc, id, ot, tm, mh, tn, dm, mf, dv, kf, fx, dq, nm, ox, px, rx, ui, sy] |
| 12 | conference abstract*.mp. [mp=ti, ab, hw, tc, id, ot, tm, mh, tn, dm, mf, dv, kf, fx, dq, nm, ox, px, rx, ui, sy] |
| 13 | historical article*.mp. [mp=ti, ab, hw, tc, id, ot, tm, mh, tn, dm, mf, dv, kf, fx, dq, nm, ox, px, rx, ui, sy] |
| 14 | 9 or 10 or 11 or 12 or 13 |
| 15 | adult.mp. [mp=ti, ab, hw, tc, id, ot, tm, mh, tn, dm, mf, dv, kf, fx, dq, nm, ox, px, rx, ui, sy] |
| 16 | (8 and 15) not 14 |
| 17 | limit 16 to human |
| 18 | limit 17 to "300 adulthood <age 18 yrs and older>" |
| 19 | limit 18 to english language |
| 20 | limit 19 to "0110 peer-reviewed journal" |
| 21 | remove duplicates from 20 |
