# Supplementary material for: Health outcomes in Deaf signing populations: A systematic review
Source: PLoS One. 2024 Apr 16;19(4):e0298479. doi: 10.1371/journal.pone.0298479 (PMC11020444; doi:10.1371/journal.pone.0298479)
Supplement: Attachment — Submitted filename: Response to Reviewers.docx [file pone.0298479.s004.docx]

**PONE-D-24-02887**

**Health outcomes in Deaf signing populations: a systematic review**

**PLOS ONE**

**Response to Academic Editor comments**

| **Academic Editor comments** | **My responses** | **Location of changes made** |
| --- | --- | --- |
| Please ensure that your manuscript meets PLOS ONE's style requirements, including those for file naming. The PLOS ONE style templates can be found at  https://journals.plos.org/plosone/s/file?id=wjVg/PLOSOne_formatting_sample_main_body.pdf and  https://journals.plos.org/plosone/s/file?id=ba62/PLOSOne_formatting_sample_title_authors_affiliations.pdf | Thank you for this. I can confirm that the manuscript body formatting now follows the required guidelines. | Throughout |
| **Funding statement:**  Please provide an amended statement that declares *all* the funding or sources of support (whether external or internal to your organization) received during this study, as detailed online in our guide for authors at http://journals.plos.org/plosone/s/submit-now.  Please also include the statement “There was no additional external funding received for this study.” in your updated Funding Statement.  Please include your amended Funding Statement within your cover letter. We will change the online submission form on your behalf. | The sentence “There was no additional external funding received for this study.” has now been added in the funding statement.  This has been amended in the cover letter. | Pg. 4., line 71. |
| Please provide a complete Data Availability Statement in the submission form, ensuring you include all necessary access information or a reason for why you are unable to make your data freely accessible. If your research concerns only data provided within your submission, please write "All data are in the manuscript and/or supporting information files" as your Data Availability Statement. | The data availability statement now includes the statement "All data are in the manuscript and/or supporting information files" |  |
| Please include captions for your Supporting Information files at the end of your manuscript, and update any in-text citations to match accordingly. Please see our Supporting Information guidelines for more information: http://journals.plos.org/plosone/s/supporting-information. | ‘Supporting information’ captions have been added at the end of the manuscript. The in-text citations have now been included. | Pg., 4, line 61 & 65; Pg. 7, line 133 & 140; Pg.47, line 720-722. |

**Response to Reviewer #1 comments**

| **Reviewer #1 comments** | **My responses** | **Location of changes made** |
| --- | --- | --- |
| **Introduction**  The introduction sets the stage but may lack a compelling narrative that highlights the nuances of health disparities faced by Deaf communities. It could benefit from integrating a broader range of literature to contextualize the unique health challenges and communication barriers Deaf people encounter. Additionally, specifying the research question or hypothesis at the outset could help sharpen the focus. | We have added an opening statement about the focus of the systematic review to the introduction to sharpen the focus for the reader. The research questions remain in the following section.  Additional emphasis concerning disparities in the access to and navigation of the health care system and its likely detrimental consequences for Deaf patients has been added. | Pg. 5, line 75-77.  Pg. 6, line 102-117. |
| **Methods**  While following PRISMA guidelines lends credibility, the methodology section could improve by offering more clarity on the database search strategy, including search terms and inclusion/exclusion criteria. A detailed explanation of the study selection process (e.g., screening and eligibility assessment) and the rationale behind the chosen quality assessment tools for evaluating the included studies would enhance transparency and replicability. | Thank you for pointing out that we were not clear about this process. The heading ‘Eligibility criteria’ has been changed to ‘search strategy’. Search terms, keywords and the operators applied in the search are now included in the S1 table. The inclusion/exclusion table 1 is now correctly identified as part of the screening and study selection process and the subheading ‘Study selection’ has been added to make it easier for the reader. The rationale for using quality assessment tools to assess bias has been explained in the quality assessment subheading. | Pg. 7, line 135  Pg.7, line 142  Pg. 9, line 163-165 |
| **Results**  The results section is crucial for understanding the scope of health disparities among Deaf individuals. However, it could be more user-friendly. For instance, employing tables or figures to summarize the study characteristics and main findings could aid in visualizing the data. Discussing the heterogeneity of the study populations and the interventions assessed would also be valuable. A more nuanced examination of the results, considering different types of health outcomes (physical vs. mental health) and the quality of the evidence, is needed. | The results section has now been expanded to include more information such as the numbers of records identified for each stage of screening, and a more detailed explanation of the study characteristics. The description of the study sample (e.g. number of participants; mean age; and gender) has been included in table 2.  With regards to interventions, not all identified studies focus on the outcomes of specific interventions. Table 2 provides details of the interventions where applicable.  Two new tables have now been included: study characteristics, and the findings of each study, which includes listing the health outcomes, for ease in visualising the data in table formats. | Pg. 9/10, line 170-174; line 177-180.  Pg. 10, line 186.  Pg. 18, line 224. |
| **Discussion**  The discussion adeptly identifies gaps in the research and potential implications. However, it could delve deeper into the mechanisms driving the observed disparities, such as access to healthcare, communication barriers, or socioeconomic factors. A comparison with other minority groups facing similar health disparities could offer additional insights. Moreover, a stronger emphasis on actionable recommendations for stakeholders at various levels (e.g., healthcare systems, policy, community engagement) would make this section more impactful. | The discussion has now been expanded to address, socioeconomic factors and the rights of Deaf people. However, the systematic review never set out to address evidence on the cause of any disparities recorded in the studies about health outcomes. It set out to assess the state of the evidence available on health outcomes in this population and the potential strengths and weaknesses of that evidence. We have been cautious, give the fact this is a formal systematic review, about drawing conclusions for stakeholders, clinicians and communities. | Pg. 37, line 466-473.  Pg. 38, line 482-493. |
| **Conclusion**  The conclusion succinctly wraps up the review but could be more forceful in calling for action. Highlighting specific, actionable steps that different sectors can take to address the disparities identified could provide a clear path forward. Additionally, emphasizing the importance of inclusive research practices that involve Deaf communities in the creation of knowledge about their health could inspire more community-engaged research efforts. | Whilst we would agree with the reviewer about the importance of inclusive research practices and a ‘call for action’, we have sought to ensure that our conclusion remains with the usual boundaries of a formal systematic review which would indicate that the primary call for action must be about increasing the quality of the evidence available on health outcomes. We have therefore included some key points about study designs that are missing and limitations of the methodologies of some of the studies identified withing the discussion. | Pg. 39, line 515-520. |
| **Overall**  General Observations for Improvement: Integrating more qualitative studies into the review could offer deeper insights into the lived experiences of Deaf individuals, providing a richer context for the quantitative findings. Additionally, addressing the potential for publication bias and discussing the implications of the findings in the context of current health policy debates would enhance the article's relevance and applicability. | Thank you for your comments. This review is about health outcomes, rather than the causal mechanisms that create health inequality outcomes. Discussing the reasons for these outcomes, including risky health behaviours, would need a different review. We have however raised additional issues of lack of Deaf experience in the nature of the evidence available in the discussion section above. |  |
